# Prevalence, Predictors, and Outcomes of Resistant Hypertension in Egyptian Population

**DOI:** 10.5334/gh.1211

**Published:** 2023-06-14

**Authors:** Mohamed Khalfallah, Ayman Elsheikh, Ahmad Eissa, Basma Elnagar

**Affiliations:** 1 Assistant professor of cardiovascular medicine, cardiovascular department, faculty of Medicine, Tanta University, EG; 2 Lecturer of endocrinology, internal medicine department, faculty of Medicine, Tanta University, EG; 3 Lecturer of cardiovascular medicine, cardiovascular department, faculty of Medicine, Tanta University, EG

**Keywords:** prevalence, predictors, outcomes, resistant hypertension, Egyptian population

## Abstract

**Background:**

Hypertension is a leading problem; it affects around thirty million adult Egyptians, according to the last national registry. The exact prevalence of resistant hypertension (RH) in Egypt wasn’t spotted before. The purpose of this study was to determine the prevalence, predictors, and impact on adverse cardiovascular outcomes among adult Egyptians with RH.

**Methods:**

The present study examined a cohort of 990 hypertensive patients who were divided into two groups based on their blood pressure control; group I (n = 842) patients who achieved blood pressure control and group II (n = 148) patients who met the RH definition criteria. All patients underwent a close follow-up for one year to evaluate the major cardiovascular events.

**Results:**

The prevalence of RH was 14.9%. The main predictors impacting the cardiovascular outcomes of RH were advanced age (≥65 years), the presence of chronic kidney diseases, a BMI ≥ 30 kg/m^2^, and NSAID use. After one year of follow-up, the RH group displayed noticeably higher rates of major cardiovascular events, including new-onset atrial fibrillation (6.8% vs. 2.5%, P = 0.006), cerebral stroke (4.1% vs. 1.2%, P = 0.011), myocardial infarction (4.7% vs. 1.3%, P = 0.004), and acute heart failure (4.7% vs. 1.8%, P = 0.025).

**Conclusion:**

The prevalence of RH in Egypt is moderately high. Patients with RH have a far higher risk of cardiovascular events than those whose blood pressure is within control.

## Introduction

Hypertension (HTN) is a major reversible clinical obstacle leading to increased morbidity and mortality globally. It affects approximately 1.3 billion people and is responsible for 7.5 million deaths a year [1]. Despite the progress in effective medical treatment, there has been a doubling in the incidence of HTN worldwide, especially in low and middle-income countries with a deficiency in controlling blood pressure (BP) [2]. According to the USA registry, 47% of adults have hypertension, and 24% of patients with hypertension aren’t controlled [3]. The burdened cost was estimated at 131 billion dollars in the USA each year [4]. In an Egyptian registry, 29.2% of the adult population had hypertension, and only 27.1% achieved controlled BP [5,6].

Resistant hypertension (RH) is a subtype of hypertension characterized by the difficulty of achieving BP goals below 140/90 mmHg despite the maximal dose of three anti-hypertensive medications, including diuretics, or achieving BP goals on four anti-hypertensive medications [7]. The clinical characteristics of patients with RH in comparison with those with non-resistant hypertension are that they tend to be older, black, obese, diabetic, and have a history of chronic kidney disease [8]. The definite cause is unknown, however, there are several impending mechanisms contributing to the development of RH, including increased sodium and fluid retention, increased activity of the sympathetic nervous system, enhancement of aldosterone levels, and the renin-angiotensin system, leading to arterial stiffness, myocardial fibrosis, and vascular remodelling [9].

Uncontrolled BP is associated with a higher increase in all cardiovascular morbidity and mortality [7]. Each 10 mmHg rise in BP was linked to a higher risk of sudden cardiac death [10]. Also, SPRINT (Systolic Blood Pressure Intervention Trial) revealed the significant profits on mortality amongst individuals with high cardiovascular risk who achieved a powerful reduction in their BP [11]. Early recognition of patients with RH is paramount for further investigations and tailoring the management. A meta-analysis study on 3.2 million patients estimated the global prevalence of RH at 10.3% [12], although that prevalence has a wide variant range due to its multifactorial nature depending on genetics, demographical, and socioeconomic factors. In Egypt, the precise incidence of RH is indistinct. The current study was constructed to be the first to evaluate the prevalence of resistant hypertension among the Egyptian population. And also, to highlight its contributing factors and outcomes to intensify prevention and management and consequently reduce the burden of raised BP on national health.

## Patients and Methods

The current study was conducted on patients who visited the cardiology outpatient clinic at Tanta university hospital with a confirmed diagnosis of hypertension between May 2020 and September 2021. The diagnosis of HTN was established either by the previous history of diagnosis and treatment of HTN or by blood pressure ≥ 140/90 mmHg on 2 to 3 office visits two weeks apart, besides their home BP >130/80 mmHg or a single BP measurement of ≥180/110 mmHg. The sample size was calculated using the EPI 7 ™ info program (CDC, Atlanta, USA) [13] with a 99.9% confidence interval, a 5% margin of error, and according to Naseem et al. [14], who reported a 12% prevalence of resistant hypertension. The minimum representative sample size was estimated to be 620, but this was doubled 1240 to account for the non-response or drop-out rates. The final number to be analyzed was 990 subjects.

According to the control of blood pressure, the patients were divided into two groups: group I (controlled hypertension) included 842 patients who achieved blood pressure measurements < 140/90 on two different visits, and group II (resistant hypertension) included 148 patients whose BP was ≥140/90 mmHg, despite being adherent to medications and taking 3 antihypertensive drugs on the optimal dose, including the diuretic, or taking four or more drugs, nevertheless they weren’t controlled. All patients who enrolled in the study signed informed consent. The study was accepted by the Ethical Committee of Tanta Medical Faculty and agreed with the principles of the Declaration of Helsinki II.

At baseline, a detailed history was taken for risk factors evaluation, including age, sex, smoking, diabetes, dyslipidemia, physical activity, excess use of salt diet >5 g sodium per day, previous history of cardiovascular, cerebrovascular, chronic kidney, and peripheral arterial diseases, obstructive sleep apnea, family history of hypertension, and history of their medications, which include antiplatelets, statins, chronic use of nonsteroidal anti-inflammatory drugs (NSAIDs) if taken more than three times per week, antidepressants, and corticosteroid drugs. In addition, the dose of antihypertensive drugs, and their adherence to prescribed medication were assessed by a questionnaire with every single visit. Moreover, the patients’ level of education, income category, place of residence, and presence of health insurance were assessed. Consequently, occupational and marital status were asked.

A physical examination was done for all patients, including an assessment of their weight, height, body mass index (BMI), heart rate, blood pressure, and ankle-brachial index. Blood pressure measurements at baseline were average for the second and third visits after the established diagnosis of HTN. All patients underwent laboratory investigations, an electrocardiogram (ECG) was done for all patients at baseline, at the end of 12 months, and at any cardiovascular events. Transthoracic echocardiography was carried out for all patients using the Vivid E9 ultrasound system (GE Vingmed Ultrasound, Horten, Norway) equipped with an M5S phased array transducer (2.5–5.0 MHz) according to the guidelines of the American Society of Echocardiography [15]. Two-dimensional, M mode, 2D Doppler, and tissue Doppler modalities were utilized for providing left ventricular ejection fraction (LVEF), left atrial volume index (LAVI), peak velocity of mitral early diastole (E) and late diastole (A) waves, E/A ratio, the average peak of the early diastolic myocardial velocity of septal and lateral walls (E’) and E/E’ ratio measurements. Also, carotid intimal media thickness (IMT) was performed by the same machine using a 9L-D linear transducer (2.4–10 MHz), at 1 cm proximal to the bifurcation of the common carotid artery.

Secondary causes of hypertension were excluded, including renal artery stenosis, coarctation of the aorta, and endocrine abnormalities, before patients› enrolment. the compliance to prescribed medication and its doses was checked during the first visit and the scheduled follow-ups at the 2nd and 4th weeks from the first visit then after 3, 6, 9, and 12 months using a questionnaire at every single visit. Patients with non-compliance or non-adherence to medication were excluded from the study. Patients who were absent during follow-ups, experienced an acute systemic illness, such as COVID-19 were also excluded from the study. We used both home and office BP monitoring to rule out the white-coat effect and patients with white coat hypertension were excluded from the study. At each visit, blood pressure and heart rate were measured, along with clinical evaluations for recording and assessing the occurrence of any major cardiovascular events. The primary endpoint of the study was the occurrence of mortality or major adverse cardiovascular events (MACE), including cardiac events (myocardial infarction, unstable angina, acute heart failure, and new-onset atrial fibrillation), cerebral events (stroke, transient ischemic attack, and cerebral haemorrhage), resuscitation after cardiac arrest, and acute peripheral vascular ischemia.

## Statistical analysis

Statistical analysis was performed using SPSS 23, IBM, Armonk, NY, USA. Quantitative data were expressed as mean ± standard deviation. Qualitative data were expressed as frequency and percentage. The student’s t-test was used to test the significance between the two groups in the quantitative data. A chi-square (X^2^) test was used to assess two qualitative parameters. A two-sided P value > 0.05 was considered statistically significant. Multivariate regression analysis was done to identify the independent predictors of RH.

## Results

The current study included 990 patients with hypertension divided into two groups: 842 patients (85.1%) had a controlled BP <140/90 mmHg (group I), and 148 patients (14.9%) had RH (group II). Baseline characteristics, risk factors, and socioeconomic factors of all patients in both groups were summarized in Table 1. Overall, the age was higher in group II than in group I (62.94 ± 10.89 vs. 61.01 ± 11.02 years, P = 0.049), with no significant difference in sex between the two groups. Patients with RH had a higher prevalence of chronic kidney diseases (27.7% vs. 20.0%, P = 0.033), obstructive sleep apnea (9.5% vs. 5.2%, P = 0.043), and obesity (28.4% vs. 24.4%, P = 0.030). Also, they showed a higher use of NSAIDs (23% vs. 16.2%, P = 0.042), a lower level of education and a higher salt intake in their diet.

**Table 1:** Basal characteristics, risk factors and socioeconomic factors of all patients in both groups.


	GROUP I (N = 842) (CONTROLLED HTN)	GROUP II (N = 148)(RESISTANT HTN)	P. VALUE

Age, years	61.01 ± 11.02	62.94 ± 10.89	0.049*

Male gender, n (%)	437 (51.9%)	80 (54.1%)	0.629

Smoking, n (%)	299 (35.5%)	49 (33.1%)	0.572

Diabetes, n (%)	261 (31.0%)	51 (34.5%)	0.403

Dyslipidemia, n (%)	307 (36.5%)	52 (35.1%)	0.757

Cardiovascular diseases, n (%)	159 (18.9%)	30 (20.3%)	0.692

Chronic Kidney diseases, n (%)	168 (20.0%)	41 (27.7%)	0.033*

Cerebrovascular diseases, n (%)	80 (9.5%)	19 (12.8%)	0.212

Peripheral vascular diseases, n (%)	102 (12.1%)	24 (16.2%)	0.167

Obstructive sleep apnea, n (%)	44 (5.2%)	14 (9.5%)	0.043*

Obesity, n (%)	172 (20.4%)	42 (28.4%)	0.030*

Family history of high blood pressure, n (%)	271 (32.2%)	48 (32.4%)	0.953

Lack of physical activity, n (%)	448 (53.2%)	86 (58.1%)	0.270

Atrial fibrillation, n (%)	109 (12.9%)	25 (16.9%)	0.196

Non-steroidal anti-inflammatory drugs use, n (%)	136 (16.2%)	34 (23.0%)	0.042*

Antidepressant drugs use, n (%)	50 (5.9%)	6 (4.1%)	0.360

Corticosteroid drugs use, n (%)	52 (6.2%)	12 (8.1%)	0.378

Cholesterol lowering medication use, n (%)	442 (52.5%)	72 (48.6%)	0.388

Antiplatelet medication use, n (%)	377 (44.8%)	64 (43.2%)	0.730

Diuretics, n (%)	601 (71.4%)	148 (100%)	0.001*

Beta-blocker, n (%)	535 (63.5%)	98 (66.2%)	0.532

ACE inhibitors OR ARB, n (%)	513 (60.9%)	133 (89.9%)	0.001*

Calcium channel blocker, n (%)	495 (58.8%)	127 (85.8%)	0.001*

Others, n (%)	245 (29.1%)	42 (28.4%)	0.859

History of non-adherence to medication, n (%)	297 (35.3%)	65 (43.9%)	0.044*

Marital status, married, n (%)	495 (58.8%)	88 (59.5%)	0.878

Income category, low income, n (%)	477 (56.7%)	89 (60.1%)	0.430

Level of education, lower, n (%)	369 (43.8%)	78 (52.7%)	0.045*

Residence, urban, n (%)	481 (57.1%)	82 (55.4%)	0.697

Occupational status, employed, n (%)	450 (53.4%)	77 (52.0%)	0.750

Health insurance, n (%)	347 (41.2%)	60 (40.5%)	0.878

Excessive salty diet, n (%)	409 (48.6%)	90 (60.8%)	0.006*


*: Significant P value, ACE: angiotensin converting enzyme; ARB, angiotensin receptor blockers.

As regarding the clinical, laboratory, and echocardiographic data for both groups. The RH group had a higher BMI (29.18 ± 3.38 kg/m^2^) than the other group (26.72 ± 4.45 kg/m^2^). The RH group had significantly elevated systolic and diastolic blood pressure values (159.5 ± 17.3 vs. 129.4 ± 11.6 mmHg and 95.20 ± 8.76 vs. 80.59 ± 8.34 mmHg, respectively, P = 0.001). However, the discrepancies in glycemic and lipid profiles between the two groups were negligible. Raised serum creatinine was substantially greater in the RH group, with a decrease in e-GFR in relation to the controlled BP group (1.25 ± 0.60 vs. 1.13 ± 0.50 mg/dl, P = 0.012) and (89.09 ± 15.0 vs. 92.09 ± 17.2 mL/min/1.73 m^2^, P = 0.047), respectively. Microalbuminuria, serum potassium, uric acid, ankle-brachial index, and carotid IMT levels did not differ significantly between the two groups as shown in Table 2.

**Table 2:** Clinical, laboratory findings and echocardiographic data of all patients in both groups.


	GROUP I (N = 842) (CONTROLLED HTN)	GROUP II (N = 148)(RESISTANT HTN)	P. VALUE

BMI, (kg/m^2^)	26.72 ± 4.45	29.18 ± 3.38	0.001*

Heart rate, (bpm)	79.74 ± 15.2	81.99 ± 18.0	0.107

Systolic BP, mmHg	129.4 ± 11.6	159.5 ± 17.3	0.001*

Diastolic BP, mmHg	80.59 ± 8.34	95.20 ± 8.76	0.001*

Fasting plasma glucose (mg/dl)	118.5 ± 17.2	117.1 ± 16.6	0.362

2-h post prandial plasma glucose (mg/dl) (mg/dl) (mmol/L)	167.8 ± 31.1	168.6 ± 40.5	0.784

HbA1c %	6.354 ± 1.26	6.428 ± 1.28	0.513

Hemoglobin, g/dl	12.05 ± 1.06	12.07 ± 1.05	0.825

TSH (mlU/L)	4.31 ± 1.91	4.53 ± 2.85	0.233

Total cholesterol (mg/dl)	223.9 ± 38.8	224.0 ± 45.1	0.982

Triglycerides (mg/dl)	165.8 ± 32.3	163.6 ± 27.6	0.428

LDL (mg/dl)	137.4 ± 27.0	141.3 ± 30.1	0.111

HDL (mg/dl)	43.68 ± 7.34	44.33 ± 7.93	0.324

Serum creatinine (mg/dl)	1.13 ± 0.50	1.25 ± 0.60	0.012*

e-GFR (mL/min/1.73 m^2^)	92.09 ± 17.2	89.09 ± 15.0	0.047*

Albuminuria (mg/g)	29.01 ± 5.01	29.12 ± 4.04	0.791

Serum potassium (mmol/L)	4.49 ± 0.79	4.48 ± 0.79	0.945

Uric acid (mg/dl)	5.69 ± 1.07	5.58 ± 1.30	0.250

Ankle brachial index	1.04 ± 0.14	1.02 ± 0.13	0.140

LVEDD (cm)	5.75 ± 0.51	5.71 ± 0.57	0.326

LVESD (cm)	3.85 ± 0.43	3.78 ± 0.44	0.088

LVEF, (%)	62.76 ± 3.77	62.54 ± 4.48	0.524

LVH, n (%)	395 (46.9%)	88 (59.5%)	0.005*

E/A	1.37 ± 0.42	1.39 ± 0.42	0.631

E/E’	11.5 ± 1.36	11.8 ± 1.03	0.019*

LAVI (ml/m^2^)	33.6 ± 1.70	34.1 ± 3.04	0.016*

LVMI (gr/m^2^)	115.2 ± 27.1	122.7 ± 25.3	0.002*

Carotid IMT (mm)	0.99 ± 0.11	0.98 ± 0.10	0.274


BMI: body mass index, BP: blood pressure, HbA1c: glycated hemoglobin, TSH: thyroid stimulating hormones, LDL: low density lipoprotein, HDL: high density lipoprotein, e-GFR: estimated glomerular filtration rate, LVEDD: left ventricle end-diastolic dimensions, LVESD: left ventricle end-systolic dimensions LVEF: left ventricle ejection fraction, LVH: left ventricular hypertrophy, E: peak early diastolic velocity, A: peak late diastolic velocity, E’: peak early diastolic myocardial velocity, LAVI: left atrium volume index, LVMI: left ventricular mass index, IMT: intima media thickness, *: Significant P value.

Concerning echocardiographic findings, E/E’ was significantly higher in the RH group (11.8 ± 1.03 vs. 11.5 ± 1.36, P = 0.019), and LAVI was noticeably higher P = 0.016. Furthermore, the percentage of patients with LVH was raised in the RH group in comparison to the controlled BP group (59.5% vs. 46.9%, P = 0.016) with a higher LVMI (122.7 ± 25.3 vs. 115.2 ± 27.1 gr/m^2^, P = 0.002).

**Figure 1:**
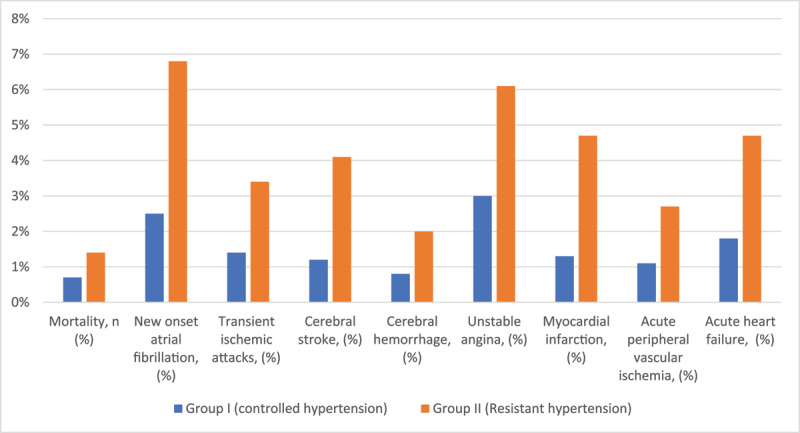
Major cardiovascular events of both groups after one year of follow up.

The major cardiovascular events that occurred in all participants were demonstrated in Table 3 and Figure 1. Patients with RH had a higher incidence of new-onset atrial fibrillation (6.8%), myocardial infarction (4.7%), acute heart failure (4.7%), and cerebral stroke (4.1%) at the end of the research. In terms of mortality or other cardiovascular events, there was no discernible difference. In multivariate regression analysis for detecting the independent predictors affecting cardiovascular outcomes in RH, the main predictors were: age ≥ 65 years (OR = 5.449; 95% CI, 2.237–13.274; P = 0.001), chronic kidney diseases (OR = 5.083; 95% CI, 2.111–12.240; P = 0.001), body mass index ≥ 30 kg/m^2^ (OR = 3.095; 95% CI, 1.339–7.153; P = 0.008), and NSAIDs use (OR = 5.681; 95% CI, 2.585–12.487; P = 0.001), as illustrated in Table 4.

**Table 3:** Major cardiovascular events of both groups after one year of follow up.


	GROUP I (N = 842) (CONTROLLED HTN)	GROUP II (N = 148)(RESISTANT HTN)	P. VALUE

Mortality, n (%)	6 (0.7%)	2 (1.4%)	0.423

New onset atrial fibrillation, n (%)	21 (2.5%)	10 (6.8%)	0.006*

Transient ischemic attacks, n (%)	12 (1.4%)	5 (3.4%)	0.092

Cerebral stroke, n (%)	10 (1.2%)	6 (4.1%)	0.011*

Cerebral hemorrhage, n (%)	7 (0.8%)	3 (2.0%)	0.180

Unstable angina, n (%)	25 (3.0%)	9 (6.1%)	0.055

Myocardial infarction, n (%)	11 (1.3%)	7 (4.7%)	0.004*

Acute peripheral vascular ischemia, n (%)	9 (1.1%)	4 (2.7%)	0.107

Acute heart failure, n (%)	15 (1.8%)	7 (4.7%)	0.025*


*: Significant P value.

**Table 4:** Multivariate regression analysis showing the independent predictors affecting cardiovascular outcomes.


	MULTIVARIATE ANALYSIS		P. VALUE

	OR	(95% CI)	

Age ≥ 65 years	5.449	2.237–13.274	0.001*

Chronic Kidney diseases	5.083	2.111–12.240	0.001*

Obstructive sleep apnea	1.809	0.473–6.910	0.386

Body mass index ≥ 30 kg/m^2^	3.095	1.339–7.153	0.008*

History of non-adherence to medication	1.439	0.418–4.955	0.564

Non-steroidal anti-inflammatory drugs use	5.681	2.585–12.487	0.001*

Lower level of education	1.687	0.591–4.815	0.328

Excessive salty diet	2.540	0.911–7.079	0.075


*: Significant P value.

## Discussion

Resistant hypertension is a challenge in controlling the BP and management of hypertension-related complications, resulting in a remarkable increase in cardiovascular diseases, end organ damage, and premature death. Hereditary genes, aberrant sympathetic and renin-angiotensin-aldosterone system activation, endothelial dysfunction, decreased arterial compliance, and increased systemic vascular resistance all contribute to the pathogenesis of resistant hypertension [7,16]. Subsequently, it heightened the need for more research to study the nature of the disease and its corresponding factors to decrease its burden on socioeconomic states, even in high-income countries. However, the precise predominance of RH in Egypt is unspecified due to limited studies. Accordingly, the present study was established in a tertiary center in the Delta region of Egypt to detect the prevalence, underlying factors, and major adverse cardiovascular outcomes of RH in the Egyptian population.

The prevalence of RH was 14.9% in the current study. The incidence of RH differed around the world and was estimated at around 10–20% [17]. In-depth, it was around 6.5% in the UK [18], 9.9% in Spain [19], 19.7% in the USA [8], and 11% in Brazil [20]. Furthermore, a meta-analysis study testified an incidence of 12.1% in Africa [21]. Also, the prevalence was reported by 14.3% from Lesotho [22], 19.0% from Algeria [23], 8.6% from Ethiopia [24], and 9.4% from the Democratic Republic of the Congo [25]. This discrepancy is substantial, which could be attributed to the variance in patient demographics, medicinal interventions, and clinical characterisation.

Comparable to the age of the patients in the present study, patients with RH were significantly older than the controlled BP group (62.94 ± 10.89 vs. 61.01 ± 11.02 years, P = 0.049). In agreement, many studies demonstrated a significant increase in RH incidence with aging [8,9,23], which was explained by an increase in vascular remodelling and resistance due to loss of arterial wall elasticity, besides an increase in multi-morbidity factors owing to poor BP control. Chia et al. [26], on the other hand, showed no significant difference between both groups regarding the age of the patients.

The current study revealed that the number of patients suffering from CKD was higher in the RH group. In correspond with these results, a meta-analysis demonstrated the high prevalence of RH in CKD (29%) and renal transplantation patients (56%) [12]. In parallel, several studies found a significant link between RH to CKD and low e-GFR [8,27,28,29], while it is hard to distinguish whether CKD was the primary cause or the secondary cause of RH. That was illustrated by the activation of the renin-angiotensin-aldosterone system and endothelin-1, combined with a decrease in nitric oxide level, with the consequences of decreased renal blood flow and renal injury [30].

The prevalence of obesity and BMI was significantly higher in patients with RH in the current study. These results came in agreement with Gijón-Conde et al. [19], Naseem et al. [14], and Bangalore et al. [31]. Furthermore, Egan et al. [32] studied 13,375 hypertensive patients and reported that BMI ≥30 kg/m^2^ doubled the possibility of RH; moreover, visceral obesity was attached to a high aldosterone level with a final result of uncontrolled BP. In the present study, RH was linked to obstructive sleep apnea (P = 0.043). Supporting this result, several studies, including a large cohort study in the USA, demonstrated an increase in the intensity risk of RH with obstructive sleep apnea by 60% [14,33,34]. By further analysis, the higher use of NSAIDs was observed among the RH patients. In addition, Naseem et al. [14] and Buhnerkempe et al. [28] remarked on their raised use, owing to the high incidence of osteoarthritis with aging. An excessive salty diet was common in the RH group (60.8%), which was known for its direct effect on raising BP and counteracting the response to antihypertensive drugs. In parallel, the reduction of dietary salt had implications for the reduction of BP [7].

Concerning echocardiographic findings, there was a noticeable increase in the incidence of LVH and LVMI in patients with RH. In concordance with the present results, Dobrowolski et al. [35] revealed a significant rise in LVMI and the prevalence of concentric hypertrophy (33%), in 155 patients with RH. Furthermore, Cao et al. [36] extended their findings to involve studying the prevalence of eccentric LV remodeling in refractory hypertension in addition to the prevalence of concentric remodeling in RH. Moreover, in the assessment of diastolic function, the RH patients had significantly higher E/E’ and LAVI, which indicated a greater reduction in left ventricular diastolic function among RH patients. In resemblance, Cao et al. [36] disclosed a higher incidence of diastolic dysfunction amongst the RH group than the non-resistant group.

The RH group had a poorer outcome after one year of follow-up, and MACE rates were significantly higher in this group of patients. The RH group had a considerably greater incidence of new-onset atrial fibrillation (6.8%) than the controlled group (2.5%). It is the consequence of high E/E’ and LAVI, resulting in left atrial remodeling and an increase in its filling pressure. Additionally, acute heart failure incidence was higher in the RH group (4.7% vs. 1.8%). Heart failure with preserved ejection fraction was previously known to be the predominant type in RH, which may be explained by the current results of an increase in LA filling pressure and a reduction of diastolic function [37].

Additionally, cerebral stroke was (4.1%) in the RH group and (1.2%) in the controlled BP group. Likewise, the myocardial infarction rate was significantly elevated in the RH group. Nevertheless, no significant difference in mortality could be detected owing to the short follow-up period. Similar to the previous results, Chun et al. [38] after 4.5 median years of follow-up of the RH patients, demonstrated a substantial rise in MACE, besides non-fatal cardiovascular events, acute HF hospitalization, and renal events, with no significant increase in death. Also, in a large retrospective study of 3.8 years of median follow-up that included approximately 200,000 patients, the RH patients were significantly accompanied by an increase in total MACE (18.0% versus 13.5%, P < 0.001) [39].

Concerning the independent predictors influencing the cardiovascular outcomes in the present study, a multivariate regression analysis was done, showing the following predictors: age ≥ 65 years, CKD, BMI ≥ 30 kg/m^2^, and the use of NSAIDs. In the ALLHAT Trial, old age, BMI ≥ 30 kg/m^2^, and serum creatinine above 1.5 mg/dl were linked to failure to reach BP goals, a high incidence of atherosclerosis, and stiffness in the vessels [40]. Therefore, patients with RH had high cardiovascular morbidity and mortality. Moreover, Thomas et al. [41] studied 3367 patients with CKD and RH and reported that increasing the degree of obesity (BMI ≥ 30 kg/m^2^) was an independent factor associated with a high risk of RH, and patients with CKD were at a higher risk of increased cardiovascular events, especially heart failure. Also, the use of NSAIDs was combined with an increase in the prevalence of RH, especially in the elderly and CKD patients, due to deterioration of kidney function, renal ischemia, and activation of the renin-angiotensin systems [42].

## Limitation

The present study was a one-center study with only one-year follow-up, and a quietly small number of populations, to a certain extent, reflected the prevalence of RH in Egypt. As a result, multicenter studies on RH are needed to identify the prevalence of RH in other geographical areas in Egypt, such as Upper Egypt, and to augment awareness of the disease’s hazardous complications as well as the importance of lifestyle adjustments, and regular follow-ups.

## Conclusion

Resistant hypertension is a heading socioeconomic burden on global health, especially in growing countries like Egypt. Its prevalence is rising and differs according to the demographic characterization of the populations. It was an adjunct to the significant risk of MACE. So, early identification of the affected patients and establishing a proper modifiable plan of management are crucial, along with reliable follow-up. Furthermore, a special interest in geriatric, obese, NSAIDS users, and CKD patients is prudent to reduce the progress of end-organ damage and MACE.
